# Preventing complications in cardiac pacemaker therapy: a lifecycle-based risk management framework

**DOI:** 10.3389/fcvm.2026.1849223

**Published:** 2026-06-01

**Authors:** Yue Li, Rui Li, Jun Zhang, Xin Yang, Luhui Gan, Yu Liao, Hua He, Lingyun Jiang, Xin Zhang

**Affiliations:** 1 Department of Cardiology, The People’s Hospital of Rongchang District, Chongqing, China; 2 Department of Oncology, The People’s Hospital of Rongchang District, Chongqing, China; 3 Department of Cardiology, West China Hospital, Sichuan University, Chengdu, China

**Keywords:** cardiac pacemaker, device infection, lead complications, lifecycle risk management, pacemaker complications, risk stratification

## Abstract

Pacemaker implantation remains an established therapeutic strategy for a broad range of bradyarrhythmias and conduction system disorders. As implantation volumes continue to increase worldwide, device-related complications have emerged as significant determinants of patient outcomes, healthcare utilization, and long-term prognosis. Pacemaker-related complications demonstrate substantial heterogeneity in terms of temporal onset, underlying pathophysiological mechanisms, and clinical impact. These events may arise from procedural factors, lead or device dysfunction, device-associated infection, or subsequent structural and functional alterations of the heart. This review proposes a structured lifecycle-based risk management framework that integrates complication prevention across the entire continuum of pacemaker therapy. This approach highlights the dynamic nature of complication risk across different phases of device therapy. Within this lifecycle risk-management paradigm, complication prevention should extend beyond isolated perioperative measures and encompass structured preprocedural assessments, standardized intraoperative techniques, and longitudinal postprocedural surveillance. By synthesizing current evidence regarding the underlying mechanisms and clinical manifestations of pacemaker-related complications and contemporary prevention and management strategies for these complications, this work provides a comprehensive overview. This framework-based approach offers a practical strategy to enhance risk stratification, guide procedural decision-making, and improve long-term outcomes in patients undergoing pacemaker therapy.

## 1 Introduction

Pacemaker implantation has become a cornerstone therapy for bradyarrhythmias and conduction system disorders, with rapidly increasing implantation volumes worldwide (1, 2). With this global increase in implantation volume, the efficacy of pacemakers in relieving bradycardia-related symptoms and improving quality of life has been well established (1–3). Nevertheless, pacemaker-related complications remain clinically significant, with incidence varying across patient populations and procedural settings, and contributing to reintervention, adverse outcomes, and increased healthcare utilization (4–6). These complications span a broad temporal spectrum—from the periprocedural period to long-term follow-up—and are characterized by heterogeneous clinical presentations and multifactorial pathogenesis. Rather than arising from a single determinant, pacemaker-related complications reflect the dynamic interplay of device characteristics, procedural factors, patient-specific susceptibility, and longitudinal management practices (4–6). Different complication types across distinct temporal stages are associated with different risk profiles and clinical contexts: infection-related events are more strongly influenced by baseline patient characteristics and periprocedural management, whereas noninfectious complications are more closely associated with device-specific and procedural factors (7–9). However, existing studies have largely focused on individual complication types or isolated clinical scenarios, and a comprehensive, lifecycle-oriented synthesis of pacemaker-related complications remains lacking. Accordingly, this review proposes a structured “lifecycle risk management” paradigm, conceptualizing complication prevention and control as a continuous and dynamic process spanning the entire treatment continuum. We systematically synthesize complication types, key risk determinants, and intervention strategies across three critical phases: preprocedural assessment, intraprocedural management, and postprocedural follow-up. On the basis of this framework, we further propose a structured risk management model aimed at enhancing long-term safety and clinical benefit in real-world pacemaker therapy.

This narrative review was informed by a structured search of the PubMed database up to January 3, 2026, aiming to identify key clinical guidelines, consensus statements, systematic reviews, and high-quality observational studies relevant to pacemaker-related complications and their prevention. The search strategy included the following keywords: “pacemaker implantation”, “pacemaker-related complications”, “lead-related complications”, “device-related infection”, and “prevention strategies”. Foundational studies addressing key pathophysiological mechanisms were included without time restriction to provide conceptual context. Evidence selection and synthesis were based on clinical relevance and conceptual importance, providing a comprehensive, framework-oriented integration of the available literature.

**Figure 1:**
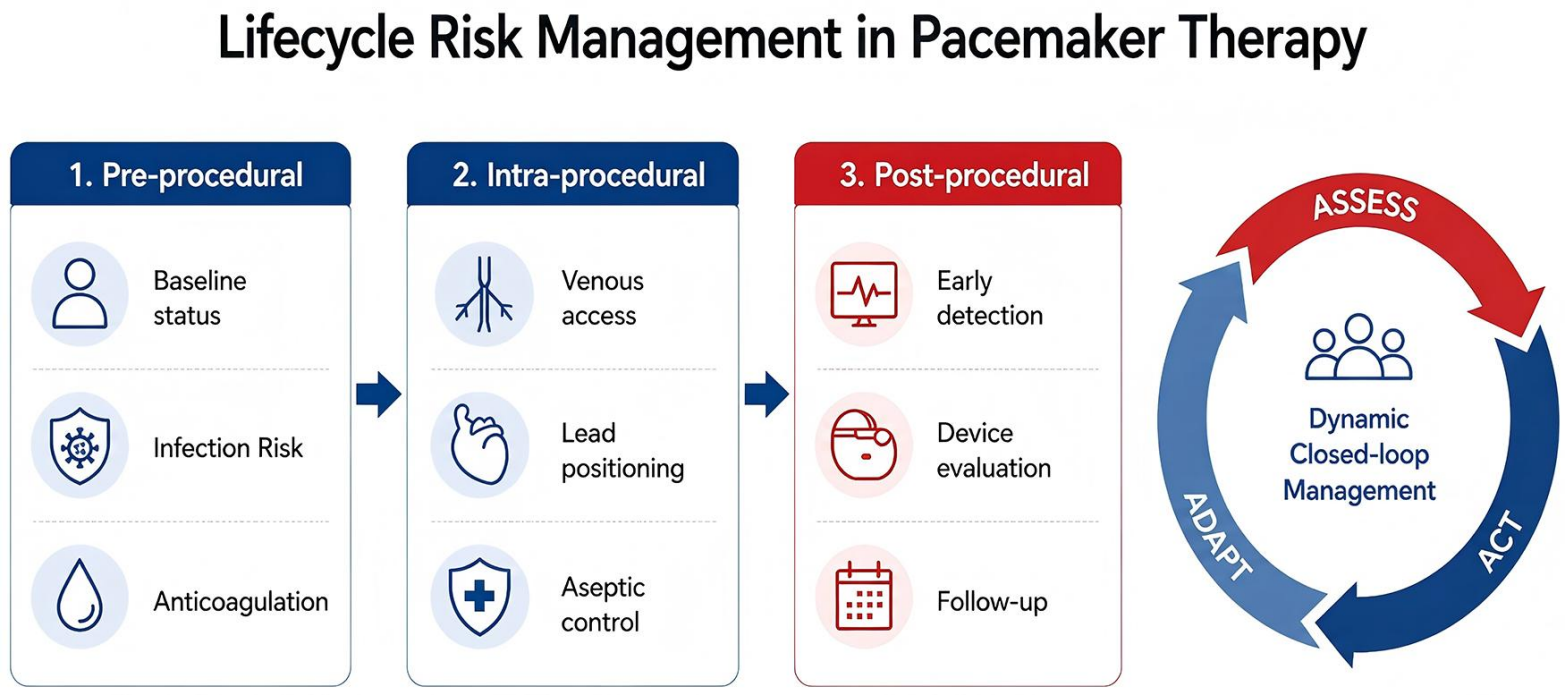
Lifecycle-based risk management framework for pacemaker therapy.

While this review focuses specifically on permanent pacemaker therapy, certain aspects—particularly device-related infection, remote monitoring, and lead extraction—draw on evidence derived from a broader group of cardiac implantable electronic devices (CIEDs). Given the shared procedural techniques and underlying pathophysiological mechanisms of CIEDs, these findings are interpreted within the clinical context of pacemaker practice. The conceptual framework of lifecycle-based risk management in permanent pacemaker therapy is shown in Figure 1. It integrates phase-specific risk stratification, procedural standardization, and structured longitudinal surveillance within a dynamic closed-loop model. To our knowledge, this is the first review to conceptualize pacemaker complication prevention within a structured lifecycle-based framework.

## 2 Classification of pacemaker-related complications

Pacemaker-related complications demonstrate distinct temporal patterns and mechanistic heterogeneity. Establishing a comprehensive lifecycle risk management framework requires the systematic categorization of complications according to both their temporal distribution and underlying pathophysiological mechanisms. Traditional classification systems have largely described complications according to individual event categories. However, different complications frequently exhibit temporal overlap and share underlying mechanisms in real-world clinical practice. Accordingly, this section systematically reorganizes pacemaker-related complications to account for both temporal and mechanistic perspectives, clarifies potential patterns of interrelationship between these complications, and provides a structured foundation for subsequent risk stratification and proactive intervention strategies. A structured classification of pacemaker-related complications according to temporal pattern, core mechanisms, representative events, and management focus is summarized in Table 1.

**Table 1:** Classification of pacemaker-related complications.

Category	Temporal pattern	Core mechanisms	Representative events	Key management focus	Evidence level/evidence basis
Peri-procedural complications	Intraoperative and early post-implant period	Procedural trauma vascular injury anticoagulation imbalance technical factors	Pneumothorax pocket hematoma/ bleeding cardiac perforation	venous access strategy appropriate anticoagulation management lead positioning standardized procedural workflow	Guideline [ESC 2021 (1)] + Observational studies (4, 5) + RCT (anticoagulation management) (45)
Lead-related complications	Early or late occurrence	Lead material and structural properties fixation mechanism mechanical stress lead–cardiac interaction	Lead dislodgement conductor fracture insulation failure Sensing/pacing threshold abnormalities lead-related perforation tricuspid regurgitation	Anatomically guided implantation tension control structured device follow-up timely intervention	Guideline [ESC 2021 (1)] + Observational study (18)
Device-related infection	risk persists throughout the device lifecycle	Bacterial adherence biofilm formation repeated invasive manipulation	Pocket infection lead-related infection device-related endocarditis	Risk stratification optimized antimicrobial prophylaxis hematoma prevention structured wound surveillance	Guideline [ESC 2021 (1), EHRA 2020 (8)] + Meta-analysis (7)
Long-term and functional complications	Gradual development during long-term follow-up	Cumulative structural changes electromechanical dyssynchrony chronic lead–tissue interaction	Venous stenosis/occlusion tricuspid regurgitation pacemaker syndrome pacing-induced cardiomyopathy	Remote monitoring reduction of right ventricular pacing burden parameter optimization early intervention window management	Guideline [ESC 2021 (1)] + Observational studies (30, 32, 33)

### 2.1 Perioperative complications

Perioperative complications occur predominantly during pacemaker implantation or in the early postoperative period and represent the earliest recognized pacemaker-related complications (4, 5). The most common perioperative complications include pneumothorax, pocket hematoma, and clinically significant bleeding requiring intervention (5). Evidence from systematic reviews and prospective studies indicates that different venous access routes are associated with differences in complication type and risk (10, 11). A previous randomized controlled trial (RCT) demonstrated that the perioperative anticoagulation strategy significantly influences the risk of pocket hematoma, underscoring the importance of careful management in patients undergoing continued or interrupted anticoagulation (12). Multivariate analyses from large cohort studies have identified anticoagulation therapy, a low body mass index, female sex, a lower annual operator volume, and increased procedural complexity as independent predictors of bleeding-related complications, including pocket hematoma (4, 13). Although cardiac perforation is relatively uncommon, it is associated with the lead type, fixation mechanism, implantation site, and myocardial structural characteristics, with intraoperative lead manipulation and mechanical stress potentially contributing (14, 15). Overall, perioperative complications are influenced by not only device- and technique-related factors but also procedural standardization and operator experience (4, 13).

### 2.2 Lead-Related complications

Global surveys indicate that most pacemaker implantation procedures involve transvenous, bipolar, and active-fixation leads (16). Follow-up studies have indicated that lead-related complications remain relatively common and include lead dislodgement and fracture, insulation defects, abnormalities in sensing or pacing thresholds, cardiac perforation and valvular dysfunction, all of which may compromise long-term device performance (17). Real-world studies suggest that lead-related complications may occur in the early postimplantation period or develop progressively during long-term follow-up and represent important causes of lead dysfunction and reintervention (5). Lead dislodgement is among the most common early lead-related complications (17, 18). Regression analyses have shown that lead dislodgement is associated with pacing or sensing dysfunction and may require reintervention in some cases (18). Lead fracture and insulation defects typically occur several years after implantation; these defects are associated with lead design, insulation material, and manufacturer and often manifest as pacing parameter abnormalities or impedance changes, sometimes remaining asymptomatic until detection during device interrogation (18, 19). The interaction between leads and cardiac structures constitutes an additional source of complications, manifesting as lead-related cardiac perforation or valvular structural and functional abnormalities resulting from chronic mechanical interference (20, 21). Studies indicate that leads traversing the tricuspid valve are associated with an increased risk of tricuspid regurgitation, particularly in older patients, those with a thinner myocardial wall, or those receiving specific lead types, and that early symptoms are often nonspecific, making timely recognition challenging (20–23). Clinically, lead-related complications often require repeated procedures, including lead revision, replacement, or extraction, increasing the perioperative risk and long-term management burden (18). Device replacement and system upgrades are also associated with a substantial risk of serious complications and increased inpatient resource use (6, 18). Despite continuous improvements in lead materials and fixation techniques, lead-related complications remain common in high-risk populations and during long-term follow-up and continue to pose a substantial clinical challenge in pacemaker therapy (5, 16–18).

### 2.3 device-related infections

Pacemaker-related infection is among the most serious and management-challenging complications of device implantation, occurring either early after implantation or during long-term follow-up, with risk persisting throughout the device lifespan; this type of complication is associated with increased reintervention rates, greater healthcare utilization, and elevated mortality (8, 24). Depending on the extent of involvement, infection may present as a localized pocket infection, a lead-related infection, or device-related infective endocarditis involving intracardiac structures (8). Predisposing factors for infection include patient-related comorbidities such as diabetes mellitus, chronic kidney disease, chronic obstructive pulmonary disease (COPD), malignancy, and heart failure, as well as clinical factors such as skin disorders, preoperative fever, and exposure to corticosteroids or anticoagulation therapy (7, 8). Procedure-related factors are likewise independently associated with increased infection risk, particularly in the context of device replacement or upgrade; lead revision; greater procedural complexity; perioperative hematoma formation; prolonged operative duration; limited operator experience; preoperative temporary pacing; and the absence of guideline-recommended antibiotic prophylaxis (7, 8, 25, 26). The pathophysiological basis of device-related infection is thought to involve bacterial adhesion to the device surface following invasive manipulation, with subsequent biofilm formation that confers increased tolerance to antimicrobial therapy and facilitates persistent infection (27). Perioperative pocket hematoma has been identified as a key intermediary factor in the pathogenesis of device-related infection, because it may create a favorable microenvironment for bacterial colonization and subsequent biofilm formation, thereby significantly increasing infection risk (28, 29).

### 2.4 Late structural and functional complications

In addition to perioperative and device-related complications, pacemaker implantation is associated with late structural and functional sequelae, including venous stenosis or occlusion, tricuspid regurgitation, pacemaker syndrome, and pacing-induced cardiomyopathy (21, 30, 31). These complications often develop insidiously and present with nonspecific clinical manifestations; however, their progressive and cumulative nature can substantially compromise long-term prognosis and quality of life (5). With respect to venous access complications, venous thrombosis and stenosis are relatively common after transvenous lead implantation and generally stabilize within six months (30). These events are thought to be related to lead-induced vascular injury and venous stasis and, in a subset of patients, may progress to severe stenosis or complete occlusion, occasionally manifesting as upper extremity edema or pulmonary embolism (30). With respect to structural complications, chronic mechanical interference by transvalvular leads is recognized as a key mechanism contributing to the development or progression of tricuspid regurgitation (32, 33). In addition, atrioventricular dyssynchrony induced by right ventricular pacing may lead to functional (“pseudo”) tricuspid regurgitation, primarily resulting from atrial contraction against a closed tricuspid valve; notably, this form of regurgitation may be partially or completely reversible following the restoration of atrioventricular synchrony (34, 35). In long-term follow-up studies, persistent lead-related tricuspid regurgitation has been independently associated with increased risks of mortality and heart failure-related events (36). With respect to functional complications, pacing-induced cardiomyopathy and pacemaker syndrome suggest that heart rate support alone is insufficient to ensure sustained long-term clinical benefit (37). Randomized trials and real-world studies have further demonstrated that a high right ventricular pacing burden is associated with increased risks of heart failure hospitalization, atrial fibrillation, and pacing-induced cardiomyopathy, thereby strengthening the evidence linking cumulative right ventricular pacing exposure to adverse cardiac remodeling and dysfunction (38, 39). Accordingly, the insidious and progressive nature of these late structural and functional complications underscores the need for structured long-term follow-up and supports a proactive management strategy centered on dynamic device optimization and early intervention.

### 2.5 Summary

In summary, pacemaker-related complications are diverse in type and demonstrate substantial heterogeneity in both temporal onset and clinical impact, encompassing not only perioperative technique-related events but also late structural and functional sequelae (4–6). This temporal and mechanistic complexity underscores the need for a structured classification framework to facilitate early identification of high-risk populations and critical procedural phases. Grounded in the “lifecycle-based risk management” paradigm, complication management should move beyond the reactive treatment of established events toward proactive intervention during early risk evolution. By integrating systematic risk stratification, procedural optimization, and structured long-term follow-up, management can be shifted upstream, ultimately improving long-term outcomes for patients with permanent pacemakers.

## 3 Lifecycle-Based framework for the prevention of pacemaker-related complications

Given the substantial heterogeneity of pacemaker-related complications in terms of timing, underlying mechanisms, and clinical impact, their prevention and management cannot be effectively addressed through isolated or phase-limited interventions. Instead, a comprehensive longitudinal approach is required. Accordingly, we propose a lifecycle-based risk management paradigm that conceptualizes pacemaker therapy across three interconnected phases: preprocedural assessment, intraprocedural management, and postprocedural follow-up. This framework emphasizes the dynamic evolution and interdependence of risk factors over time, recognizing complications as the cumulative result of continuous, multifactorial processes rather than discrete events. Within this paradigm, systematic risk stratification and patient optimization are prioritized in the preprocedural phase, complemented by standardized procedural execution and key technical safeguards during implantation, followed by structured long-term follow-up with dynamic surveillance. This integrated approach facilitates upstream risk mitigation and ensures continuity of care across the therapeutic trajectory. In addition, the model incorporates a closed-loop management strategy—comprising risk identification, targeted intervention, outcome evaluation, and iterative adjustment—thereby shifting the traditional paradigm from reactive, event-driven management toward proactive, risk-oriented prevention. Collectively, this lifecycle-based framework provides a coherent conceptual foundation for integrating prevention strategies across different complication types and supports the implementation of phase-specific, evidence-informed interventions, consistent with current guideline-driven approaches to continuous risk management across the device lifecycle (8). To enhance the clinical applicability of the proposed lifecycle-based framework, validated risk stratification tools can be incorporated into the preprocedural phase to enable quantitative assessment of patient-level risk. Among these, the PADIT score, a validated risk prediction model established in multicenter studies, provides a structured and evidence-based approach for estimating the risk of CIED infection, thereby supporting risk-adapted preventive and procedural strategies across subsequent phases of the device therapy lifecycle (Table 2).

**Table 2:** PADIT-based risk stratification for device-related infection.

Domain	Predictor/risk category	Category/definition	Score/outcome
Scoring system	P - Prior procedures	0 procedures	0
		1 procedure	1
		≥2 procedures	3
	A - Age	≥70 years	0
		60–69 years	1
		<60 years	2
	D - Depressed renal function	eGFR < 30 mL/min/1.73 m^2^	1
	I - Immunocompromised status	Presence of immunosuppression	3
	T – Type of procedure	Pacemaker (implantation/replacement)	0
		ICD (implantation/replacement)	2
		CRT (implantation/replacement)	4
		Revision/upgrade (pocket/lead/system)	4
Risk stratification	Low risk	PADIT score 0–4	1-year infection rate: 0.51%
	Intermediate risk	PADIT score 5–6	1-year infection rate: 1.42%
	High risk	PADIT score ≥7	1-year infection rate: 3.41%

## 4 Prevention and management of pacemaker-related complications

Given the substantial variability in the timing, mechanisms, and clinical consequences of pacemaker-related complications, prevention and management of these complications necessitate a structured framework that extends beyond isolated or phase-specific interventions to encompass preoperative assessment, intraoperative optimization, and postoperative follow-up, guided by risk identification and complication-specific stratification (1). The available evidence indicates that the preventability and optimal timing of intervention vary across complication types, supporting a stage-specific and risk-adapted strategy aimed at preserving the benefits of pacemaker therapy while minimizing both immediate and long-term adverse effects (1, 4, 5). Accordingly, the following sections outline current prevention and management approaches across three key phases: preoperative evaluation, intraoperative standardization, and postoperative surveillance.

### 4.1 Preoperative risk assessment and patient selection

A comprehensive and structured preoperative risk assessment constitutes a foundational component in minimizing pacemaker implantation-related complications and optimizing long-term clinical outcomes (1). The determinants of adverse events are inherently multifactorial, encompassing both patient-specific and procedural factors. At the patient level, advanced age, a low body mass index (BMI), and a high burden of comorbidities have consistently been associated with elevated complication rates, with different predictive factors observed across short-term and long-term follow-up periods (4, 5, 7, 8). At the procedural level, device complexity, the type of procedure, and institutional and operator experience further modulate risk, with complex interventions—such as reimplantation procedures and system upgrades—being associated with a substantially higher incidence of complications than *de novo* implants are (6). Accordingly, the central objective of preoperative evaluation is not only risk identification but also the integration of these variables into a structured stratification framework that guides individualized perioperative planning. In patients deemed high risk, the optimization of comorbidity management, the implementation of tailored procedural strategies, and engagement in multidisciplinary collaboration when appropriate may collectively attenuate the likelihood of adverse events and improve overall safety profiles.

### 4.2 Intraoperative technical optimization and procedural standardization

Intraoperative procedural quality, encompassing critical steps such as venous access, lead implantation, and device fixation, represents a pivotal determinant of perioperative complication risk (1). Nationwide registry data and real-world analyses consistently demonstrate a strong association between operator experience and device-related complication rates (4, 40).

During venous access, both the choice of entry site and the use of imaging guidance directly influence early adverse events, including pneumothorax and vascular injury (41). Systematic reviews and meta-analyses have shown that ultrasound-guided axillary vein access reduces vascular access-related complications without compromising procedural success while also decreasing fluoroscopy exposure (10, 42). A randomized controlled trial further confirmed the feasibility and safety of ultrasound-guided axillary vein access, providing higher-level evidence to support the incorporation of this technique into standardized vascular access protocols (11). In the lead implantation phase, the lead type, the fixation mechanism, and implantation site selection are closely linked to the risks of lead dislodgement and myocardial perforation (14, 20, 43). Anatomically guided positioning, together with meticulous fixation and appropriate tension control, constitutes a key technical strategy for minimizing lead-related reinterventions (14, 20, 43). Infection prevention represents another essential component of intraoperative standardization. A prospective study demonstrated that major device infections are associated with increased mortality and represent a substantial healthcare burden (44). Given these serious consequences, intraoperative infection control should be reinforced through the use of strict aseptic technique, appropriate antimicrobial prophylaxis, meticulous hemostasis, and intensified preventive measures in high-risk scenarios (41). Collectively, effective prevention of perioperative complications depends on the systematic integration of optimized venous access, refined implantation techniques, rigorous infection control, and coordinated team-based procedural workflows rather than reliance on any single technical modification.

### 4.3 Perioperative anticoagulation management and pocket hematoma risk control

Pocket hematoma represents a key intermediary linking perioperative anticoagulation strategies to subsequent device-related infection, whereas anticoagulation management itself directly determines the risk of hematoma formation (8, 28). Accordingly, effective hematoma prevention represents a key leverage point in reducing downstream infectious complications. Standardized perioperative anticoagulation management is therefore central to an integrated prevention framework for pacemaker-related complications. The BRUISE CONTROL trial demonstrated that compared with heparin bridging, continuation of anticoagulation therapy during device implantation significantly reduced pocket hematoma after warfarin interruption in patients receiving warfarin (45). In contrast, the BRUISE CONTROL-2 trial revealed that among patients treated with direct oral anticoagulants (DOACs), relative to temporary interruption of anticoagulation therapy, continuation of anticoagulation therapy did not result in a reduction in hematoma risk (46). Collectively, the current evidence supports an individualized anticoagulation strategy based on a balanced assessment of thromboembolic and bleeding risks. Decision-making should incorporate the type of anticoagulant agent, procedural complexity, and patient-specific characteristics, with the aim of avoiding unnecessary interruption or bridging while maintaining adequate protection against thromboembolism (45, 46).

### 4.4 Infection prevention and standardized management

Pacemaker-related infection is among the most clinically severe and resource-intensive complications of device therapy and is strongly associated with repeated procedures, increased healthcare utilization, and increased long-term mortality (8, 24, 47). Current evidence and international guidelines uniformly recommend the implementation of infection prevention across the entire perioperative continuum—the preoperative, intraoperative, and postoperative phases—because deficiencies at any stage may substantially increase cumulative risk (8). During the preoperative phase, patient-related risk factors—including diabetes mellitus (DM), chronic kidney disease (CKD), heart failure (HF), chronic obstructive pulmonary disease (COPD), and malignancy—should be systematically evaluated and optimized, and prophylactic systemic antibiotics should be administered within one hour before skin incision to ensure adequate tissue concentrations at the time of implantation (7, 8, 48, 49). Intraoperative prevention focuses on minimizing tissue trauma, ensuring meticulous hemostasis and layered closure, performing appropriate pocket irrigation, and, in selected high-risk or complex cases, considering adjunctive measures such as the use of antibiotic-eluting envelopes to reduce bacterial colonization and subsequent biofilm formation (8, 47, 50). Postoperative management emphasizes the early identification of local inflammatory signs and prompt standardized intervention, including careful wound care, the prevention and control of pocket hematoma, the avoidance of unnecessary hematoma aspiration, and the minimization of early reinterventions that may increase the risk of contamination (7, 8, 51). Importantly, infection typically reflects the cumulative contribution of

multiple modest risk factors rather than a single precipitating event; once a weakness in the infection prevention framework emerges, localized infection may progress to deep or systemic involvement (8). When infection involves leads or intracardiac structures, antimicrobial therapy alone is rarely curative; current guidelines recommend complete device system removal combined with systemic antimicrobial therapy as the standard management strategy, an approach supported by multicenter registry data demonstrating safety and improved outcomes (8, 47, 52, 53). Delayed or incomplete implementation of this strategy has been associated with increased morbidity, healthcare utilization, and mortality (54, 55). Accordingly, effective management of pacemaker-related infection requires an integrated approach encompassing comprehensive perioperative prevention, early recognition, and timely standardized system extraction when indicated while maintaining individualized decision-making within an evidence-based framework. A lifecycle-based, phase-specific management framework for device-related infection is summarized in Table 3.

**Table 3:** Lifecycle-based infection prevention strategies: phase-specific management.

Phase	Major risk determinants	Preventive strategies	Monitoring and early detection	Evidence level/evidence basis
Pre-procedural Phase	Diabetes mellitus Chronic kidney disease Chronic obstructive pulmonary disease Malignancy Heart failure Skin disorders Preoperative fever Corticosteroid therapy Anticoagulation therapy	Systematic infection risk assessment Optimization of comorbidities Standardized preoperative antimicrobial prophylaxis Individualized anticoagulation planning	Identification of high-risk patients through structured preoperative evaluation	Guideline [ESC 2021 (1)] + Expert consensus [EHRA 2020 (8)] + Meta-analysis (7)
Intra-procedural Phase	Device replacement or upgrade Lead revision Increased procedural complexity Prolonged operative duration Temporary pacing Perioperative hematoma formation Suboptimal antimicrobial prophylaxis Limited operator experience	Venous access optimization Strict aseptic technique Guideline-based antibiotic prophylaxis Meticulous hemostasis Layered wound closure Minimization of tissue trauma Selective use of antibacterial envelope systems in high-risk or complex cases	Workflow standardization Operator expertise optimization	Guideline [ESC 2021 (1)] + Expert consensus [EHRA 2020 (8)] + RCT (WRAP-IT trial) (71)
Early post-procedural phase	Pocket hematoma Early wound inflammation Unnecessary hematoma aspiration Early reintervention	Prevention and control of pocket hematoma Structured wound care Avoidance of unnecessary aspiration Prompt standardized intervention for suspicious local signs	Structured postoperative surveillance Controlled reintervention strategy	Guideline [ESC 2021 (1)] + Expert consensus [EHRA 2020 (8)] + Observational study (pocket hematoma as a predictor of subsequent device infection) (28)
Established infection phase	Pocket infection Lead-associated infection Device-related infective endocarditis Persistent or recurrent bacteremia Systemic inflammatory response Lead-associated endocarditis	Early extraction following diagnosis Complete device system removal Systemic targeted antimicrobial therapy	Early identification of device-related bloodstream infection Surveillance for systemic infectious progression	Guideline [ESC 2021 (1)] + Expert consensus [EHRA 2020 (8)] + Expert consensus [HRS 2017 (53)]
Long-term surveillance phase	High cumulative right ventricular pacing burden Multiple transvenous leads Prior transvenous interventions or reinterventions Chronic transvalvular lead interaction	Integration of device and cardiac functional assessment Dynamic pacing parameter optimization Risk-adapted system upgrade when indicated	Structured longitudinal surveillance Risk-triggered early intervention	Guideline [ESC 2021 (1)] + Observational studies (30, 32, 33)

### 4.5 Postoperative follow-up and long-term complication surveillance

In the long-term management of pacemaker therapy, postoperative follow-up extends beyond routine assessment of device function and plays a pivotal role in the early identification of late structural and functional complications (1, 39, 56, 57). After transvenous lead implantation, approximately 20%–50% of patients develop varying degrees of venous stenosis, often detected during follow-up imaging or preprocedural evaluation for generator replacement or system upgrade. Although lead diameter has not been consistently shown to independently predict venous obstruction, factors such as the presence of multiple leads, prior transvenous interventions, and repeated reinterventions appear to exert greater influence (58). Regarding functional complications, meta-analyses have indicated that persistent right ventricular pacing, mediated by chronic electromechanical dyssynchrony, can induce structural and functional myocardial remodeling, potentially progressing to pacemaker-induced cardiomyopathy (PICM) (59). Lead-related tricuspid regurgitation likewise develops insidiously and has been independently associated with worsening cardiac function and adverse long-term outcomes (36). Owing to their subtle onset and progressive course, these complications may be easily overlooked in the absence of systematic follow-up (58, 59). Structured imaging and functional monitoring therefore enable the early detection of venous access abnormalities, right ventricular pacing-related remodeling, and progressive valvular dysfunction at subclinical stages, thereby providing a window for timely parameter optimization or consideration of system upgrades (60–62). Standardized follow-up should integrate device status, pacing burden, and dynamic changes in cardiac structure and function to facilitate risk identification and inform early intervention decisions (8, 39, 58, 59, 62). Among functional complication-related metrics, the percentage of right ventricular pacing serves as a key evidence-based quantitative indicator and is closely associated with atrial fibrillation and heart failure outcomes (38, 63, 64). Strategies aimed at minimizing unnecessary right ventricular pacing—such as prolongation of the atrioventricular interval or activation of intrinsic conduction-promoting algorithms (e.g., managed ventricular pacing, MVP)—have demonstrated efficacy in randomized trials (65). Incorporation of a structured pathway consisting of “the identification of elevated pacing burden—parameter optimization—proactive intervention” into routine follow-up protocols supports the transition of pacemaker therapy from passive surveillance to risk-alert–guided dynamic management, ultimately enhancing long-term safety and clinical benefit. The identification pathways, structured surveillance focus, and optimal intervention windows for major late structural and functional complications are summarized in Table 4. Complementing these identification and monitoring strategies, the incidence of major pacemaker-related complications provides important epidemiological context for understanding their clinical impact. To provide a quantitative perspective on the burden of these complications across different procedural phases, the reported incidence is summarized in Table 5. These reported incidence ranges should be interpreted cautiously because estimates vary according to study design, patient population, device type, procedure type, complication definition, and follow-up duration.

**Table 4:** Late complications: identification, surveillance, and intervention window.

Late complication	Key identification clues	Structured surveillance focus	Intervention window
Venous stenosis/occlusion	Upper extremity edema Incidental detection during device evaluation Imaging evidence of venous narrowing	Periodic venous patency assessment in patients with multiple leads or prior interventions	Early intervention before complete occlusion or need for complex reintervention
Lead-related tricuspid regurgitation	Progressive valvular dysfunction Worsening heart failure symptoms	Longitudinal echocardiographic evaluation of valvular function	Early consideration of lead repositioning or system modification before irreversible right-sided dysfunction
Right ventricular pacing–related adverse remodeling	High cumulative right ventricular pacing burden Chronic electromechanical dyssynchrony Persistent atrioventricular asynchrony	Minimization of unnecessary right ventricular pacing Dynamic pacing parameter optimization Consideration of system upgrade when indicated	Before irreversible ventricular remodeling develops

**Table 5:** Complications incidence: lifecycle distribution and evidence basis.

Phase	Specific complications	Reported Incidence	Evidence level/evidence basis
Intra-procedural Phase	Pneumothorax	^∼^0.5%–2%	Guideline [ESC 2021 (1)] + Observational studies (4, 5, 17)
	Cardiac perforation	^∼^0.3%–1%	Guideline [ESC 2021 (1)] + Observational study (4)
Post-procedural Phase (early)	Lead dislodgement (early)	^∼^1%–2%	Observational studies (5, 18)
	Pocket hematoma	^∼^2%–6%	Guideline [ESC 2021 (1)] + Observational studies (5, 15, 17)
	Lead perforation (delayed)	^∼^0.3%–0.7%	Observational studies (14, 18, 20)
Post-procedural Phase (long-term)	Lead fracture/insulation failure	^∼^1%–3%	Observational study (19)
	Lead-associated tricuspid regurgitation	^∼^10%–40%	Guideline [ESC 2021 (1)] + Recent review (62)
	Venous stenosis/occlusion	^∼^20%–50%	Observational study (58)
	Pacemaker syndrome	^∼^1%–20%	Guideline [ESC 2021 (1)]
Lifecycle complication	CIED infection	^∼^1%–2%	Guideline [ESC 2021 (1)] + Meta-analysis (7) + Observational studies (4, 26)

### 4.6 Summary

In summary, the prevention and management of pacemaker implantation-related complications should be conceptualized as a continuous, lifecycle-oriented process rather than as isolated actions confined to individual procedural phases. Current evidence and international guidelines consistently highlight three foundational pillars of complication reduction: preoperative risk-stratified patient assessment, standardized intraoperative techniques, and structured long-term follow-up incorporating remote monitoring (1, 8, 41). An integrated strategy that implements coordinated and targeted interventions across different phases enables earlier risk identification and proactive management, thereby continuously enhancing the safety and long-term clinical benefit of pacemaker therapy in real-world practice.

## 5 Discussion and future directions

**Figure 2:**
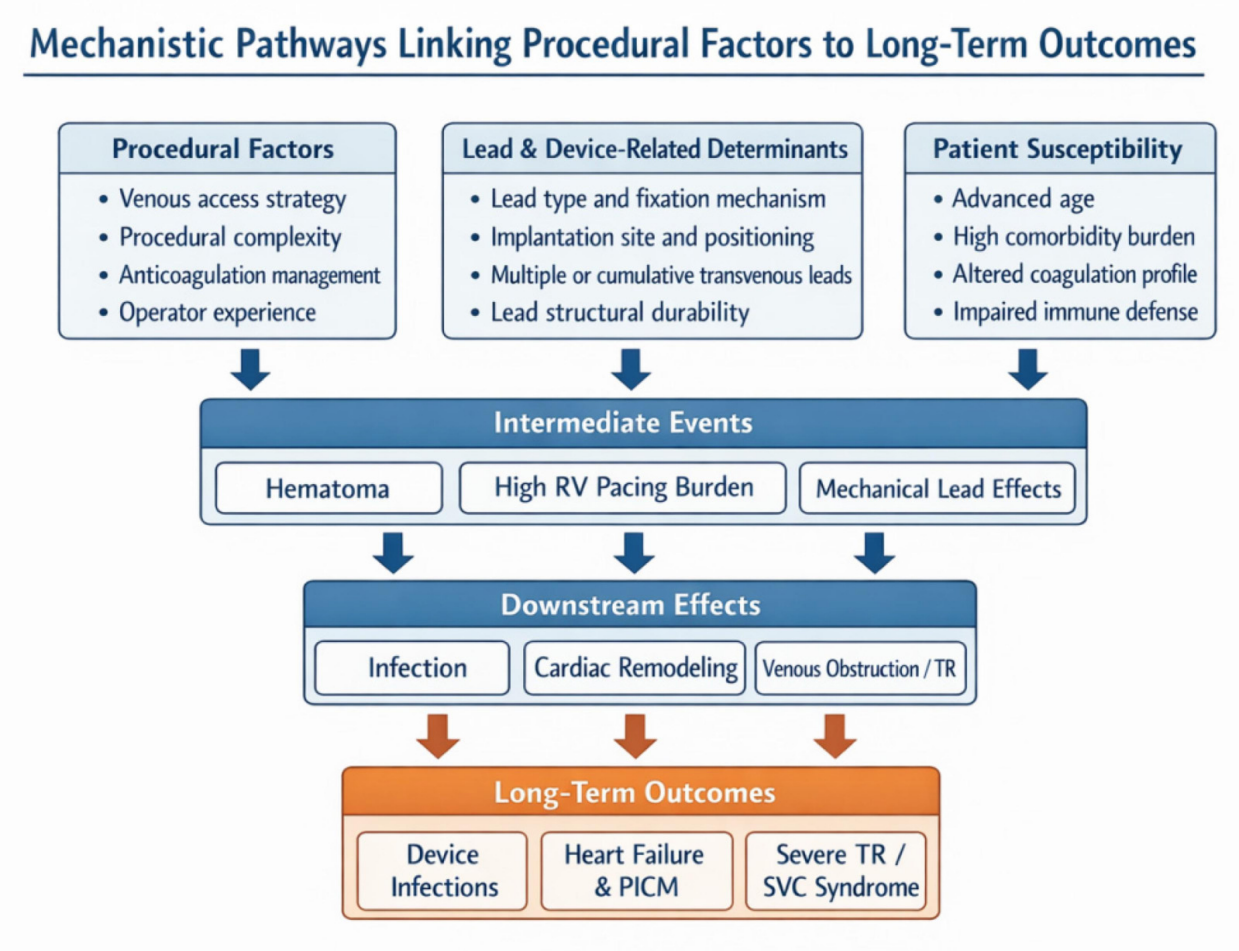
Mechanistic pathways linking procedural factors to long-term outcomes.

Despite substantial advances in implantation techniques and perioperative management, pacemaker-related complications remain an ongoing clinical challenge (1, 4, 5). The occurrence of pacemaker-related complications does not stem from any single technical or device-related deficiency but rather results from the dynamic interplay of patient baseline characteristics, operator experience, implantation strategy, device properties, and long-term management practices (1, 4). On the basis of accumulated evidence and international guideline consensus, future optimization should shift from the traditional paradigm centered on isolated technical refinements toward a comprehensive system-level transformation spanning the entire perioperative continuum—from preoperative evaluation and intraoperative management to long-term follow-up (1, 8, 41). At the device level, optimization of lead materials, enhancement of long-term structural stability, and innovation in pacing modalities constitute the foundational basis for reducing both mechanical and functional complications (1, 2). At the procedural and perioperative management levels, pathway selection guided by individual anatomical characteristics and clinical risk stratification, together with targeted management of high-risk populations, may reduce overall complication risk at its source, particularly in the context of infection prevention (1, 8). At the operator and team levels, procedural experience and the degree of workflow standardization also exert a measurable influence on complication rates (41). Accordingly, the implementation of structured training programs, the volume-based stratification of procedural practices, and continuous quality assessment can enhance procedural consistency and overall safety (40, 41). In summary, only through coordinated advancement across multiple domains—including device innovation, implantation strategy optimization, and team-based standardization—can the long-term safety and clinical benefits of pacemaker therapy be enhanced in a sustainable manner in real-world practice. Taken together, durable improvements in long-term safety and clinical outcomes depend not on isolated innovations but on coordinated progress across device engineering, procedural optimization, and team-based standardization within an evidence-informed framework. Figure 2 summarizes the mechanistic pathways linking procedural factors, lead- and device-related determinants, and patient susceptibility to intermediate events and long-term clinical outcomes.

### 5.1 Technology advances

Continuous innovation in device technology remains a fundamental strategy for mitigating pacemaker-related complications. In recent years, leadless pacing systems have introduced a new technological approach to pacing beyond the conventional transvenous paradigm, structurally reducing lead- and pocket-related complications by eliminating the need for transvenous leads and a subcutaneous pocket (66). Prospective investigations have demonstrated favorable safety and feasibility profiles, with a complication spectrum distinct from that observed with traditional systems (67). Although current indications are relatively restricted and long-term outcome data are still evolving, ongoing design refinements and expanding real-world evidence are expected to broaden the clinical applicability of these leadless pacing systems. Beyond platform-level innovations, improving the durability and functional reliability of transvenous leads remains a central priority in reducing long-term complication burden. Large-scale cohort studies have demonstrated that lead-related complications, including dislodgement, insulation failure, conductor fracture, and electrical abnormalities, are cumulative during long-term follow-up and significantly increase the need for reintervention (5, 68). Contemporary consensus statements suggest that long-term lead reliability should be incorporated into a lifecycle-oriented management framework, with strengthened efforts toward early detection and the standardized management of lead dysfunction (53). Overall, future device optimization should prioritize durable system performance while facilitating more individualized technology selection aligned with patient-specific risk profiles and clinical indications.

### 5.2 Implantation strategies

The use of standardized and precise implantation techniques constitutes a key approach for mitigating pacemaker-related complications (1, 8). With the expanding integration of image-assisted modalities—particularly ultrasound—in cardiac interventional practice, the safety and reproducibility of these modalities in pacemaker implantation have gained increasing evidence-based support. Accumulating data indicate that ultrasound-guided venous access significantly decreases puncture-related complications, reduces the incidence of pneumothorax and vascular injury, increases first-pass success rates, increases positioning accuracy, and decreases fluoroscopy time, thereby increasing overall procedural safety and efficiency (10, 11). Building on a deeper understanding of cardiac electromechanical coupling, more precise lead positioning—such as conduction system pacing—helps preserve the physiological ventricular activation sequence and mechanical synchrony and, in follow-up studies, has been associated with improved left ventricular function and favorable heart failure-related outcomes (69, 70). Collectively, the integration of image-guided techniques and physiologically informed implantation strategies not only mitigates perioperative mechanical and vascular risks but also establishes a structural and functional basis for optimizing long-term cardiac performance. As supporting evidence continues to accumulate, the incorporation of these techniques and strategies into standardized implantation protocols is expected to become increasingly central in contemporary pacing practice.

### 5.3 Infection prevention

Pacemaker-related infection remains one of the most clinically consequential and prognostically significant complications of cardiac implantable electronic devices (8, 25). Owing to the substantial heterogeneity in patient-specific and procedural risk factors, contemporary prevention strategies are increasingly transitioning from uniform prophylaxis to risk-adapted precision interventions. Risk stratification to identify high-risk patients, combined with intensified perioperative preventive strategies—including optimized antibiotic prophylaxis and the selective application of antibacterial envelope systems—may reduce the overall incidence of infection while minimizing unnecessary intervention in low-risk populations (8, 71). International consensus statements suggest that structured postoperative wound surveillance and early detection strategies, particularly in high-risk patients, facilitate timely intervention at localized stages and may prevent progression to systemic device-related infection requiring extraction (8). Therefore, infection prevention should be conceptualized as a continuous process rather than a time-limited perioperative measure. In summary, the integration of validated risk stratification models with graded preventive strategies, embedded within a longitudinal management framework spanning preoperative assessment through postoperative follow-up, represents a pivotal direction for reducing infection burden and optimizing both clinical outcomes and healthcare resource allocation.

### 5.4 Digital management

With the rapid advancement of remote monitoring systems and digital health infrastructure, pacemaker follow-up has undergone a paradigm shift from episodic, in-clinic assessments to continuous, remotely enabled, and data-driven management frameworks. This transformation extends beyond logistical convenience, fundamentally redefining how device performance and complication risk are surveilled over time. An RCT demonstrated that remote monitoring facilitates earlier identification of device-related abnormalities and clinically relevant events, leading to a shorter time to clinical intervention and a modest reduction in unplanned hospitalization rates (72). By enabling real-time data transmission and automated alerts, this approach enhances proactive clinical oversight and shifts follow-up from symptom-triggered evaluation toward data-informed intervention. From a risk management perspective, the integration of remotely acquired device data with established clinical risk factors offers a foundation for refined patient stratification. A recent study suggested that integrating remote monitoring data with clinical risk characteristics to construct predictive risk models may help identify patient subgroups that require intensified follow-up or earlier intervention, thereby supporting complication risk stratification and informed clinical decision-making (73). Although the application of artificial intelligence and machine learning in remote pacemaker management remains at an exploratory stage, these technologies hold potential as decision support tools rather than autonomous systems. Their clinical effectiveness and safety require validation in well-designed prospective studies. In the future, artificial intelligence is likely to serve as a clinical decision support tool that is integrated with remote monitoring and structured follow-up pathways to facilitate more dynamic and individualized long-term management.

### 5.5 Clinical implications

The prevention and management of pacemaker-related complications are increasingly transitioning from isolated technical refinements toward an integrated, lifecycle-oriented optimization framework encompassing device innovation, precision-guided implantation strategies, infection risk stratification, and structured long-term follow-up. Accumulating evidence indicates that no single intervention is sufficient to meaningfully reduce the overall complication burden; rather, sustained risk mitigation requires coordinated, multidimensional strategies. Advances in leadless pacing, conduction system pacing, image-guided implantation, and remote monitoring technologies have strengthened the technical foundation for safer device therapy. When integrated with validated risk assessment models and data-informed decision support systems, these innovations collectively enable a shift from reactive complication management to proactive risk governance across the treatment continuum. From a clinical perspective, this framework supports earlier risk identification, facilitates individualized procedural planning, and enables more structured long-term surveillance in real-world pacemaker practice. Through procedural standardization, risk-adapted prevention, and the strategic incorporation of emerging technologies, pacemaker therapy is progressively evolving toward a comprehensive lifecycle management paradigm, with continued refinement expected to further enhance long-term safety and clinical outcomes in real-world practice.
